# Preand post-treatment spectral-domain optical coherence tomography findings in patients with unilateral amblyopia

**DOI:** 10.5935/0004-2749.202100117

**Published:** 2021

**Authors:** Nora Lucía Oliva Castillo, Nancy Carolina Quezada del Cid, Martin Arturo Zimmermann Paiz, Ana Marissa Ordóñez Rivas, Verónica Yaneth Burgos Elías, Evelyn del Busto Wilhelm

**Affiliations:** 1 Unidad de Oftalmología Pediátrica, Estrabismo y Neuro-Oftalmología “Dra. Ana María Illescas Putzeys”, Hospital de Ojos y Oídos “Dr. Rodolfo Robles Valverde”, Instituto de Ciencias de la Visión, Benemérito Comité Pro-Ciegos y Sordos de Guatemala, Guatemala City, Guatemala

Dear editor,

Amblyopia is the most common cause of monocular visual loss in children and young adults in developed countries, and its prevalence ranges between 0.5% and 6% in the general population^(1)^.

Several reports have confirmed that amblyopia may have an effect on various components of the visual pathway, such as organic and functional changes in the geniculate nucleus and visual cortex^(2-3)^. Moreover, the examination of optic nerve head (ONH) parameters in amblyopic eyes has revealed significant changes in some of these parameters compared to those in normal eyes^(4)^.

There is limited information regarding the anatomic changes that occur in amblyopic eyes before and after occlusion treatment. Kavitha et al.^(5)^ reported no significant differences in retinal nerve fiber layer (RNFL) thickness after treatment in amblyopic eyes.

Considering the lack of conclusive data regarding the anatomic changes occurring in the visual pathway due to amblyopia or its treatment, we compared peripapillary RNFL (RNFLp) thickness and ONH anatomic features in amblyopic versus sound eyes and in amblyopic eyes before and after occlusion therapy using spectral-domain optical coherence tomography (SD-OCT) findings as an attempt to provide further information.

We included 15 patients with unilateral amblyopia with a median age of 7.3 ± 1.9 years, of whom 9 were men. In the anisometropia group (n=9), 3 patients had hyperopia, 8 patients had astigmatism, and 1 patient had hyperopia and astigmatism. In the strabismic amblyopia group (n=3), all patients had horizontal and unilateral strabismus. Regarding amblyopia severity, 1 patient had mild amblyopia and was anisometropic, 9 patients had moderate amblyopia, and 5 patients had severe amblyopia. The mean basal visual acuity values were 0.7 ± 0.3 logMAR (Logarithm of the Minimum Angle of Resolution) in amblyopic eyes and 0.15 ± 0.12 logMAR in fellow eyes (p<0.05).

The clinical features of the patients are summarized in table 1.

**Table 1:** Baseline demographic and clinical characteristics of patients

Case	Sex	Age (years)^*^	Amblyopic eye	Ocular alingment	Amblyopia severity	Amblyopic eye initial VA (logMaR)	Amblyopic eye final BCVA (logMAR)	Sound eye VA (logMaR)	Cycloplegic refractive error		Spherical equivalent (D)	
									Amblyopic eye	Sound eye	Amblyopic eye	Sound eye
1	M	9	Right	Ortotropia	Moderate	0.5	0.1	0.2	+8.50 -0.50x180°	+5.50 -0.75x180°	+8.25	+5.12
2	F	11	Left	Ortotropia	Severe	0.9	0.1	0	+2.00 -5.75x15°	+1.00	-0.87	+1.00
3	M	6	Right	Ortotropia	Moderate	0.5	0.1	0.2	+4.00 -7.00x175°	+2.25 -5.25x175°	+0.50	-0.35
4	M	5	Right	Ortotropia	Moderate	0.4	0.1	0.2	+3.00 -2.75x180°	+2.25 -1.25x180°	+1.63	+1.62
5	F	7	Left	Ortotropia	Moderate	0.5	0	0.4	+3.00 -5.00x180°	+3.50 -3.00x180°	+0.50	+2.00
6	M	10	Left	Ortotropia	Moderate	0.4	0.2	0	+2.75 -4.00x180°	+1.25 -2.50x180°	+0.75	0
7	F	10	Left	Ortotropia	Moderate	0.7	0.1	0	+3.25 -6.25x5°	+2.25 -1.00x180°	+0.12	+1.75
8	M	5	Left	Ortotropia	Moderate	0.5	0	0.1	+1.25 -3.00x10°	+1.25 -0.50x180°	-0.25	+1.00
9	F	6	Right	Ortotropia	Mild	0.3	0	0	+1.00 -1.25x180°	+0.75	+0.37	+0.75
10	M	5	Left	Ortotropia	Moderate	0.5	0.1	0.2	+1.25 -4.00x180°	+0.50	-0.75	+0.50
11	M	7	Right	Ortotropia	Severe	0.9	0	0.2	+3.25 -3.75x180°	+1.75 -1.25x180°	+1.38	+1.12
12	M	8	Left	Ortotropia	Severe	1	0.1	0.1	+1.25 -3.50x30°	+1.50 -0.25x180°	-0.50	+1.38
13	F	6	Right	RXT 30 DP	Moderate	0.5	0.1	0.2	+1.50	+1.25 -1.25x180°	+1.50	+0.62
14	F	7	Right	RET 50 DP	Severe	2	0.1	0.2	+3.50 -1.75x180°	+3.00 -1.00x180°	+2.63	+2.50
15	M	7	Left	LET 30 DP	Severe	1.3	0.3	0.4	+1.25 -3.50x180°	+1.00 -4.00x180°	-0.50	+1.00

M= male; F= female= BCVA= best corrected visual acuity; VA= visual acuity; RXT= right exotropia; RET= right esotropia; LET= left esotropia; PD= prism diopters; D= diopters.

* = At the beginning of the study

The average treatment period was 6.1 ± 4.9 months (5.5 ± 4.0 months for anisometropia and 9.0 ± 6.5 months for strabismic amblyopia, p=0.5; 5.4 ± 4.4 months for moderate amblyopia and 7.6 ± 5.7 months for severe amblyopia, p=0.5). The final visual acuity values were 0.1 ± 0.1 logMAR for patients with anisometropia and 0.2 ± 0.1 logMAR for those with strabismic amblyopia (p=0.3) and 0.1 ± 0.05 logMAR for patients with moderate amblyopia and 0.12 ± 0.1 logMAR for those with severe amblyopia (p=0.5). Patients showed improvement of an average of 4.1 ± 2.2 lines of vision; those with anisometropia showed improvement of 3.5 ± 1.4 lines, and those with strabismic amblyopia showed improvement of 6.7 ± 2.9 lines (p=0.2). Patients with moderate amblyopia showed improvement of 3.6 ± 1.5 lines of vision, and those with severe amblyopia showed improvement of 5.7 ± 3.0 lines (p=0.3).

The time taken for amblyopia resolution was 5.3 ± 4.1 months for patients with anisometropia and 9 ± 6.5 months for those with strabismic amblyopia (p=0.5). Based on the severity of amblyopia, the time taken to achieve resolution was 5.4 ± 4.4 months in patients with moderate amblyopia and 7.6 ± 5.7 months in those with severe amblyopia (p=0.5). Patient with mild amblyopia achieved resolution in 4 months.

Table 2 summarizes the comparative results of RNFLp thickness and ONH anatomic features between amblyopic and fellow eyes both before and after treatment and in amblyopic eyes. A significant difference was found only in the inferior quadrant of RNFLp thickness, which was lesser in amblyopic eyes than in fellow eyes after treatment (150.5 ± 20.9 and 159.3 ± 17.1 µm, p=0.03).

**Table 2:** Comparison of average RNFLp thickness and ONH anatomic features before and after treatment between amblyopic and fellow eyes and in anisometropic, strabismic, and moderate and severe amblyopic eyes

Parameter			
Average peripapillary NFL thickness (µm)	Amblyopic eyes (pre treatment) (n=15)	Control (fellow eyes pre treatment) (n = 15)	p value
Superior	137.4 ±17.3	136.5 ±13.4	0.8
Inferior	154.9 ±22.8	161.2 ± 16.2	0.2
Nasal	83.8 ±22	90.7 ± 11.1	0.2
Temporal	76 ± 7.8	77.1 ±11	0.6
*Total*	*113.1 ± 13.5*	*116.6 + 8.9*	*0.2*
ONH anatomic features	2.5 ±0.5	2.4 ±0.5	0.1
Optic disc area (mm^2^)	0.8 ±0.3	0.7 ±0.3	0.1
Cup area (mm^2^)	1.6 ±0.4	1.6 ±0.4	0.5
Neural rim area (mm^2^)	0.3 ±0.1	0.3 ±0.1	0.1
C/D area ratio	0.5 ±0.1	0.5 ±0.1	0.1
Lineal C/D ratio	0.6 ±0.1	0.5 ±0.1	0.1
Vertical C/D ratio	0.2 ±0.1	0.1 ±0.1	0.5
Cup volume (mm^3^) Neural rim volume (mm^3^)	0.3 ±0.1	0.2 ±0.1	0.05
Parameter			
Average peripapillary NFL thickness (µm)	Amblyopic eyes (post treatment) (n=15)	Control (fellow eyes post treatment) (n = 15)	p value
Superior	138.3 ± 13.2	137.3±12.7	0.7
Inferior	150.5 ±20.9	159.3 ± 17.1	0.03
Nasal	85.2 ±13.3	87.5 ±12.5	0.3
Temporal	74.5 ±8.7	75.7 ±9.8	0.4
*Total*	7*12.9 ± 11.7*	*114.9 + 8.9*	*0.2*
ONH anatomic features			
Optic disc area (mm^2^)	2.5 ±0.4	2.5 ±0.5	0.8
Cup area (mm^2^)	0.8 ±0.3	0.8 ±0.3	0.8
Neural rim area (mm^2^)	1.7 ±0.3	1.7 ±0.4	0.8
C/D area ratio	0.3 ±0.1	0.3 ±0.1	0.8
Lineal C/D ratio	0.6 ±0.1	0.6 ±0.1	0.6
Vertical C/D ratio	0.5 ±0.1	0.5 ±0.1	0.4
Cup volume (mm^3^)	0.1 ±0.1	0.1 ±0.1	0.8
Neural rim volume (mm^3^)	0.3 ±0.1	0.2 ±0.1	0.1

**Anisometropic eyes (n=9)**	**Pre treatment**	**Post treatment**	**p value**	**Moderate amblyopic eyes (n = 9)**	**Pre treatment**	**Post treatment**	**p value**
**Average peripapillary NFL thickness (µm)**				**Average peripapillary NFL thickness (µm)**			
Superior	137.4 ±17.3	138.2 ±14.7	0.3	Superior	144.1 ± 14.6	142.7±12.7	0.5
Inferior	154.9 ±22.8	151.1 ±23.1	0.3	Inferior	165.8 ±13.9	164.3 ±9.04	0.4
Nasal	83.8 ±22	84.7 ±12.6	0.8	Nasal	89.1 ±11.5	89.1 ±9.7	1
Temporal	76 ±7.8	73.8 ±8.9	0.1	Temporal	81 ±9.9	77.8 ±6.3	0.2
*Total*	*113.1* ± 73.5	7 72.7 ± 72.7	*0.1*	*Total*	*120.2 + 4.7*	*118.5 ±4.5*	*0.2*
**ONH anatomic features**				**ONH anatomic features**			
Optic disc area (mm^2^)	2.5 ±0.5	2.4 ±0.5	0.8	Optic disc area (mm^2^)	2.6 ±0.5	2.7 ±0.4	0.5
Cup area (mm^2^)	0.8 ±0.3	0.8 ±0.3	0.4	Cup area (mm^2^)	0.9 ±0.4	0.9 ±0.3	0.6
Neural rim area (mm^2^)	1.6 ±0.4	1.6 ±0.2	0.3	Neural rim area (mm^2^)	1.7 ±0.3	1.8 ± 0.1	0.3
C/D area ratio	0.3 ±0.1	0.3 ±0.1	0.3	C/D area ratio	0.3 ±0.1	0.3 ±0.1	0.4
Lineal C/D ratio	0.5 ±0.1	0.6 ±0.1	0.4	Lineal C/D ratio	0.6 ±0.1	0.6 ±0.1	0.5
Vertical C/D ratio	0.6 ±0.1	0.6 ±0.1	0.4	Vertical C/D ratio	0.6 ±0.1	0.5 ±0.1	0.2
Cup volume (mm^3^)	0.2 ±0.1	0.2 ±0.1	0.5	Cup volume (mm^3^)	0.2 ±0.1	0.2 ±0.1	0.8
Neural rim volume (mm^3^)	0.3 ±0.1	0.2 ±0.1	0.5	Neural rim volume (mm^3^)	0.3 ±0.1	0.3 ±0.1	0.7
**Strabismic eyes (n=3)**	**Pre treatment**	**Post treatment**	**p value**	**Severe amblyopic eyes (n=5)**	**Pre treatment**	**Post treatment**	**p value**
**Average peripapillary NFL thickness (µm)**				**Average peripapillary NFL thickness (µm)**			
Superior	126±19.6	138.7 ±2	0.4	Superior	130 ±16.4	136.4 ±3.4	0.5
Inferior	154.7 ±2	148.3 ±5.9	0.4	Inferior	147.8±12.4	138.4 ± 11.2	0.2
Nasal	73.7 ±35.6	87.3 ±15.5	0.4	Nasal	76.2 ±28.1	83.8 ± 13.12	0.5
Temporal	79.3 ±9.1	77 ±7.1	0.4	Temporal	74.6 ±8.9	70 ±10.2	0.2
*Total*	*108.3* ± 76	*113+4.5*	*0.6*	*Total*	*107* ± 72.7	*107 + 6.8*	7
**ONH anatomic features**				**ONH anatomic features**			
Optic disc area (mm^2^)	2.6 ±0.5	2.6 ±0.1	0.8	Optic disc area (mm^2^)	2.2 ±0.5	2.2 ±0.4	1
Cup area (mm^2^)	0.8 ±0.05	0.8 ±0.2	0.6	Cup area (mm^2^)	0.7 ±0.2	0.7 ±0.2	1
Neural rim area (mm^2^)	1.8 ±0.5	1.8 ±0.3	1	Neural rim area (mm^2^)	1.5 ±0.4	1.6 ±0.3	0.8
C/D area ratio	0.3 ±0.1	0.3 ±0.1	0.9	C/D area ratio	0.3 ±0.1	0.3 ±0.1	0.4
Lineal C/D ratio	0.6 ±0.1	0.6 ±0.1	0.9	Lineal C/D ratio	0.5 ±0.1	0.5 ±0.1	0.5
Vertical C/D ratio	0.5 ±0.05	0.5 ±0.03	0.6	Vertical C/D ratio	0.6 ±0.1	0.5 ±0.05	0.4
Cup volume (mm^3^)	0.1 ±0.04	0.1 ±0.1	0.3	Cup volume (mm^3^)	0.1 ±0.1	0.2 ±0.1	0.2
Neural rim volume (mm^3^)	0.2 ±0.1	0.2 ±0.1	1	Neural rim volume (mm^3^)	0.2 ±0.1	0.2 ±0.05	0.4

Age, sex, and spherical equivalent were identified as factors that could modify RNFLp thickness and ONH anatomic features in sound and amblyopic eyes, although they showed no significant associations (p>0.05).

Our study showed no significant difference in RNFLp thickness nor in ONH anatomic features in amblyopic eyes after amblyopia treatment and between amblyopic and fellow eyes.

Further studies are required to determine whether anatomic and structural ocular alterations occur after occlusion treatment in patients with amblyopia.
